# Disruption of the *ABA1* encoding zeaxanthin epoxidase caused defective suberin layers in Arabidopsis seed coats

**DOI:** 10.3389/fpls.2023.1156356

**Published:** 2023-03-15

**Authors:** Jeongho Choi, Hyojin Kim, Mi Chung Suh

**Affiliations:** Department of Life Science, Sogang University, Seoul, Republic of Korea

**Keywords:** ABA signaling, abscisic acid, Arabidopsis, seed coat, suberin

## Abstract

Suberin, a complex polyester deposited in the seed coat outer integument, acts as a hydrophobic barrier to control the movement of water, ions, and gas. However, relatively little is known about the signal transduction involved in suberin layer formation during seed coat development. In this study, the effect of the plant hormone abscisic acid (ABA) on suberin layer formation in seed coats was investigated by characterizing mutations in Arabidopsis related to ABA biosynthesis and signaling. Seed coat permeability to tetrazolium salt was noticeably elevated in *aba1-1* and *abi1-1* mutants, but not significantly altered in *snrk2.2/3/6*, *abi3-8*, *abi5-7*, and *pyr1pyl1pyl2pyl4* quadruple mutants compared with that in the wild-type (WT). *ABA1* encodes a zeaxanthin epoxidase that functions in the first step of ABA biosynthesis. *aba1-1* and *aba1-8* mutant seed coats showed reduced autofluorescence under UV light and increased tetrazolium salt permeability relative to WT levels. *ABA1* disruption resulted in decreased total seed coat polyester levels by approximately 3%, with a remarkable reduction in levels of C24:0 ω-hydroxy fatty acids and C24:0 dicarboxylic acids, which are the most abundant aliphatic compounds in seed coat suberin. Consistent with suberin polyester chemical analysis, RT-qPCR analysis showed a significant reduction in transcript levels of *KCS17*, *FAR1*, *FAR4*, *FAR5*, *CYP86A1*, *CYP86B1*, *ASFT*, *GPAT5*, *LTPG1*, *LTPG15*, *ABCG2*, *ABCG6*, *ABCG20*, *ABCG23*, *MYB9*, and *MYB107*, which are involved in suberin accumulation and regulation in developing *aba1-1* and *aba1-8* siliques, as compared with WT levels. Together, seed coat suberization is mediated by ABA and partially processed through canonical ABA signaling.

## Introduction

The seed coat surrounds the outermost layer of the seed, and the immature seed coat is comprised of five distinctive cell layers, specifically two layers of outer integument and three layers of the inner integument. Each of the layers has its own disparate developmental paths, but all of them eventually merge into two layers of dead cells (Haughn and Chaudhury, 2005). Through and beyond maturation, the seed coat provides protection against mechanical stress, various pathogens, and herbivores to the embryo and seed itself. It also enhances germination, dormancy, and seed dispersal and transmits signals from the outside to the embryo. These roles played by the seed coat contribute to the proper maturation and preservation of the seed to the next generation (Haughn and Chaudhury, 2005; Radchuk and Borisjuk, 2014). During seed coat development, the outer integument undergoes cell wall thickening (Beeckman et al., 2000), wherein suberin, a lipophilic polyester, is deposited (Molina et al., 2006; Molina et al., 2008). Suberin in the seed coat, shown to be highly concentrated in the chalazal region, contributes to seed dormancy and imparts seed coat impermeability to tetrazolium (TZ) salts (Beisson et al., 2007). It was also recently reported that seed coat suberin forms a physical barrier against heavy metals, such as Cr3+, in the soil (de Silva et al., 2021). Suberin is mainly comprised of ester-linked glycerol, very-long-chain fatty acids (VLCFAs), ω-hydroxy fatty acids, α,ω-dicarboxylic acids, primary alcohols, and ferulate. Unlike root suberin, which is dominated by monomers of C16, C18:1, and C22, seed coat suberin contains monomers of C22 and C24 as its major components (Molina et al., 2006; Li-Beisson et al., 2013).

For the synthesis of suberin aliphatic precursors in the endoplasmic reticulum, 3-ketoacyl-CoA synthase KCS2/DAISY and KCS20 contribute redundantly to the elongation of C20 to C22 VLCFAs, and KCS9 extends the C22 to C24 VLCFAs in *Arabidopsis* roots and seed coats (Franke et al., 2009; Lee et al., 2009; Kim et al., 2013). Further, potato KCS6 is involved in potato tuber periderm suberization (Serra et al., 2009). Moreover, the levels of C18, C20, and C22 fatty acids are specifically reduced by fatty acyl-CoA reductases FAR5, FAR4, and FAR1 to form respective suberin alcohols (Domergue et al., 2010; Vishwanath et al., 2013). In addition, the conversion of fatty acids to ω-hydroxy fatty acids, mediated by the hydroxylation of ω-carbon, is catalyzed by fatty acid ω-hydroxylase CYP86A1/HORST and CYP86B1/RALPH, where the former has specificity for substrates of ≤ C18 and the latter for substrates of > C20 (Höfer et al., 2008; Compagnon et al., 2009; Molina et al., 2009). ω-Hydroxy fatty acids and α,ω-dicarboxylic acids are then activated to acyl-CoA forms, which are then acylated to generate glycerol-3-phosphate by glycerol-3-phosphate acyltransferase 5 (GPAT5) (Beisson et al., 2007; Li et al., 2007; Yang et al., 2010; Yang et al., 2012). Meanwhile, phenolic monomers, including ferulic acids, are synthesized *via* the phenylpropanoid pathway, processed in the cytoplasm. Knock-out analysis of BAHD-family genes encoding aliphatic suberin feruloyl transferase/ω-hydroxyacid hydroxycinnamoyl transferase (ASFT/HHT) revealed that ASFT contributes to the incorporation of ferulate into suberin polyester by catalyzing feruloyl-CoA transfer to ω-hydroxy fatty acids and primary alcohols (Gou et al., 2009; Molina et al., 2009). The resulting suberin monomers are secreted through the plasma membrane to be deposited inside the cell wall. ATP binding cassette transporters ABCG1, ABCG2, ABCG6, and ABCG20 contribute to suberin monomer export in roots and/or seed coats (Yadav et al., 2014; Fedi et al., 2017; Shanmugarajah et al., 2019). Arabidopsis lipid transfer protein, AtLTPI-4, is involved in the transport of long-chain (LC) and very-long-chain (VLC) suberin monomers in the *Arabidopsis* crown gall (Deeken et al., 2016), and glycosylphosphatidylinositol (GPI)-anchored lipid transfer protein 15 (LTPG15) is associated with the export of VLC suberin monomers in seed coats (Lee and Suh, 2018). Upstream of aliphatic and aromatic suberin precursor biosynthesis, seed-specific MYB9 and MYB107 activate the expression of suberization-associated genes (Lashbrooke et al., 2016; Gou et al., 2017). Moreover, MYB transcription factors such as MYB36, MYB39, MYB41, MYB53, MYB92, and MYB93 function as regulators of root endodermal suberization (Kosma et al., 2014; Cohen et al., 2020; To et al., 2020; Wang et al., 2020; Shukla et al., 2021). A NAC family transcription factor, ANAC046 was reported to regulate suberization in Arabidopsis roots (Mahmood et al., 2019).

Abscisic acid (ABA) has long been implicated in the regulation of suberization. This compound is a xanthophyll-derived phytohormone (Nambara and Marion-Poll, 2005) that is actively synthesized during the seed maturation stage and contributes to the deposition of seed storage reserves, improvements in desiccation tolerance, and achieving primary seed dormancy (Kermode, 2005). ABA biosynthesis begins in the plastids with the epoxidation of zeaxanthin to form violaxanthin, which is catalyzed by zeaxanthin epoxidase, encoded by the *ABA1* locus of *Arabidopsis* (Rock and Zeevaart, 1991). Its stimulating role was first observed in suberization of the potato disk and tissue culture upon treatment with exogenous ABA (Soliday et al., 1978). Cottle and Kolattukudy (1982) further observed the increased accumulation of suberin monomers and the induction of enzymes associated with suberization in ABA-treated tissue cultures of potato tubers. Later, the effect of ABA was detected in various suberized tissues, such as the wound-healing sites of tomato (Leide et al., 2012; Tao et al., 2016), potato tuber (Lulai et al., 2008; Boher et al., 2013), and kiwifruit (Han et al., 2017; Han et al., 2018; Han et al., 2019; Wei et al., 2020a; Wei et al., 2020b) and in *Agrobacterium*-induced tumors in *Arabidopsis* (Efetova et al., 2007). The formation of *Arabidopsis* root suberin was determined to be highly stimulated by exogenous ABA treatment, and this response was hindered with the endodermis-specific expression of *abi1-1*, which suppresses ABA signaling, suggesting the involvement of ABA signaling in the regulation of *Arabidopsis* root suberization (Barberon et al., 2016).

Despite several preceding studies on the relationship between ABA signaling and suberization, little is known about whether ABA is involved in seed coat suberization. Considering that ABA signaling and seed coat suberin both significantly contribute to proper seed maturation and dormancy, it is reasonable to speculate on the relationship between them. In this study, the seed coat permeability of an ABA biosynthetic mutant, *aba1-1* and several ABA signaling mutants was tested through TZ staining. To further investigate the consequences of *ABA1* disruption in terms of seed coat suberization, an additional T-DNA insertional mutant allele of *aba1* was isolated and named *aba1-8*. Here, UV autofluorescence observations, TZ staining, and suberin monomer analysis were performed using wild-type, *aba1-1*, and *aba1-8* seeds, which were followed by RT-quantitative PCR analysis. We revealed that the disruption of *ABA1* hinders proper seed coat suberization *via* downregulation of the expression of genes involved in suberin biosynthesis.

## Materials and methods

### Plant materials

All experiments were performed using *Arabidopsis thaliana* plants. The seed surfaces were sterilized by inverting them in 0.05% Triton X-100 in 75% EtOH for 30 seconds, followed by rinsing them in 100% EtOH 2~3 times. The sterilized seeds were stratified at 4°C for 3 days and grown on 0.6% ½ MS agar medium supplemented with 1% sucrose and then transferred to soil after 7 days if needed. The plants were grown at 23°C, with 50~60% humidity under long-day conditions (16 h light/8 h dark). The T-DNA insertional mutant line, *aba1-8* (SAIL_310_A04, At5g67030), was obtained from ABRC (Arabidopsis Biological Resource Center) and selected on ½ MS agar medium containing 10 µg mL^-1^ phosphinothricin. Homogenous mutants were isolated *via* PCR screening with the primers listed in **Table S1**, and knockout of the *ABA1* gene was verified through RT-PCR, described below. The rest of the ABA biosynthesis- or signaling-defective mutant [*aba1-1*, *QC3* (*pyr1pyl1pyl2pyl4* with Col-0 background), *QL3* (*pyr1pyl1pyl2pyl4* with L*er* background), *abi1-1*, *snrk2.2/3/6+*, *abi3-8*, *abi5-7*] seeds were obtained from Prof. Soo Young Kim (Chonnam National University).

### RNA isolation and quantitative real-time polymerase chain reaction

To verify the *aba1* T-DNA insertion knockout mutant, total RNA was isolated from 9-day-old seedlings using the RNeasy^®^ Plant Mini Kit (Qiagen, 74904) following the manufacturer’s instructions. For RT-qPCR analysis, total RNA was isolated from developing siliques at 4–10 DAF, using the modified version of the methods reported by Oñate-Sánchez and Vicente-Carbajosa (2008). Here, 550 µL extraction buffer (0.2 M LiCl, 200 mM Tris-HCl pH 8.0, 25 mM EDTA, 1% SDS) and 550 µL chloroform were added to approximately 50 mg of ground tissues, vortexed vigorously, and centrifuged for 3 minutes at RT. The supernatant was mixed with 700 µL phenol/chloroform/isoamyl alcohol (IAA; Ambion, AM9720), vortexed, and centrifuged for 3 minutes at RT to remove the remaining carbohydrates. The supernatant was mixed with the same volume of chloroform and centrifuged to additionally remove carbohydrates if needed. Next, the supernatant was mixed with a 1/3 volume of 8 M LiCl and incubated at −20°C for 1 hour to precipitate the nucleic acids. The mixture was centrifuged at 4°C for 30 minutes to retrieve the nucleic acids as pellets. The supernatant was removed completely, the pellets were dissolved in RNase free water and treated with RNase free DNaseI (Qiagen, 79254), and the sample was incubated at 25°C for 1 hour. After incubation, a 1/3 volume of phenol/chloroform/IAA was added to the mixture, vortexed, and centrifuged to remove the enzymes. The supernatant was mixed with a 1/10 volume 3 M NaoAc, pH 5.2, and two volumes of 100% ethanol, inverted, and incubated at −20°C for at least 1 hour. The mixture was centrifuged at 4°C for 20 minutes, and the pellets were washed with 70% ethanol. Washed pellets were air-dried and dissolved in RNase free water. Then, 2 µg of isolated total RNA was used to synthesize complementary DNA using GoScript Reverse Transcriptase (Promega, A5003), following the manufacturer’s instructions. The obtained cDNA was used for RT-PCR and RT-qPCR analyses. *PP2AA3* (At1g13320) was used as a reference gene for RT-PCR to normalize RNA expression levels. RT-qPCR was performed using TOPreal™ qPCR 2X Premix (SYBR Green with low ROX; Bio-Rad, RT500M) following the manufacturer’s instructions, and the expression level of each gene was normalized to that of *ACTIN7* (At5g09810). The primers were used as listed in **Table S1**.

### Seed coat characterization

For suberin autofluorescence visualization, 100~110 seeds were observed at a time under ultraviolet light (365 nm excitation) and photographed using a digital camera. For the seed coat permeability assay, wild-type and *aba1* mutant seeds were immersed in 1% (w/v) TZ red (2, 3, 5-triphenyltetrazolium chloride) solution and incubated at 30°C in the dark. Stained seeds were observed after 8 and 24 hours of incubation and photographed using a digital camera equipped with a micro-lens. To quantify the amount of formazan produced, 50 mg of seeds were immersed in 500 µL of 1% (w/v) TZ solution and incubated at 30°C in the dark for the intended amount of time. After incubation, the seeds were washed in 1 mL of DW and resuspended in 1 mL of 95% ethanol. The seeds were finely ground with mortars and pestles, and the ground seeds were collected in a 2 mL Eppendorf tube. Then, 95% ethanol was added up to 2 mL, and the sample was centrifuged for 3 minutes at 15,000 x *g*. Next, 1 mL of supernatant was transferred for the measurement of absorbance at 485 nm with a spectrophotometer (Eppendorf BioSpectrometer^®^ fluorescence). For mucilage staining, seeds were soaked in 50 mM EDTA for 2 hours in advance and incubated in 0.01% ruthenium red (w/v) in 10 mM Tris-HCl (pH 8.0) for 1-2 hours. Stained seeds were washed with 10 mM Tris-HCl (pH 8.0) and photographed with a micro-lens-equipped digital camera. For proanthocyanidin staining, seeds were incubated in 1% vanillin in a 6 M HCl solution for 24 hours, washed with DW, and photographed with a micro-lens-equipped digital camera. Three replicates of each experiment were performed for statistical analysis.

### Suberin analysis

Dried seeds were used for seed coat polyester analysis, following the method described by Lee and Suh (2018). Seeds were immersed in 80°C boiling isopropanol (25 mL g^-1^ of seeds) for 10 minutes and ground thoroughly with a mortar. Ground tissues were delipidated successively using chloroform:methanol (2:1, v/v) and chloroform:methanol (1:2, v/v) with the daily exchange of the solvent and then finally extracted with methanol. Delipidated tissues were dried under nitrogen gas and desiccated for at least 24 hours using a vacuum dessicator until a constant weight was achieved. Delipidated dry residues were depolymerized *via* the base catalysis method. Internal standards (methyl heptadecanoate and ω-pentadecalactone, 5 µg each) and 2 mL of reaction solvent (3.8 mL methanol, 1.3 mL 28% sodium methoxide in methanol, 0.9 mL methyl acetate per 6 mL of solvent) were added to the dry residues and heated for 2 hours at 60°C. After cooling at RT, 4 mL of dichloromethane, 0.5 mL of glacial acetic acid, and 1 mL of 0.9% NaCl (w/v) in 100 mM Tris-HCl pH 8.0 were added, followed by centrifugation (600 x *g*, 10 min). The lower organic phase was washed at least twice with 2 mL of 0.9% NaCl (w/v) in 100 mM Tris-HCl pH 8.0. The final extract was dehydrated over anhydrous sodium sulfate and dried under a stream of nitrogen gas. The dried sample was acetylated by dissolving it in 100 µL of pyridine and 100 µL acetic anhydride and heating it for 2 hours at 60°C. After evaporation of the solvent under nitrogen gas, derivatized suberin monomers were dissolved in 50 µL heptane:toluene (1:1, v/v) and analyzed using a GC-flame ionization detector using protocols described by Lee et al. (2009).

## Results

### *ABA1* knock-out increased permeability of *Arabidopsis* seeds

**Figure 1:**
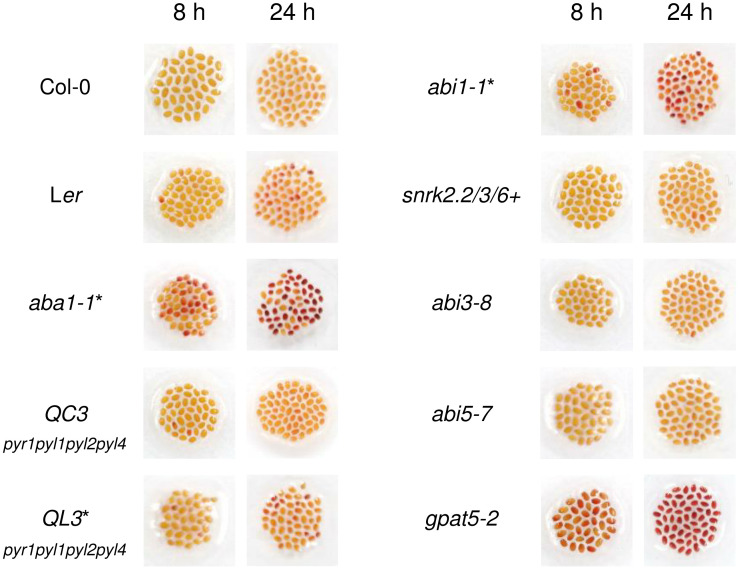
Col-0, L*er*, *aba1-1*, and several abscisic acid (ABA) signaling mutants were immersed in 1% (w/v) tetrazolium salt solution for 8 and 24 hours. After incubation, seeds were photographed with a digital camera. *gpat5-2* was used as a positive control. Those with L*er* as their background are indicated with asterisks. *QC3*, *pyr1pyl1pyl2pyl4* quadruple mutant with Col-0 background; *QL3*, *pyr1pyl1pyl2pyl4* quadruple mutant with L*er* background; *abi1-1*, PP2C mutant; *snrk2.2/3/6+*, *snrk2.2snrk2.3snrk2.6+* triple mutant.

To examine whether ABA is an important signaling molecule for the formation of *Arabidopsis* seed coat suberin, seeds of one ABA biosynthetic mutant and six different ABA signaling mutants were immersed in 1% (w/v) TZ salt for 8 and 24 h, after confirming the mutations in all tested plants except *abi3-8* and *abi5-7* (**Figure S1**). *gpat5-2*, which is defective in seed coat suberin, was used as a positive control (Beisson et al., 2007). Surprisingly, among the six ABA signaling mutants, namely *QC3*, *QL3*, *abi1-1*, *snrk2.2/3/6+*, *abi3-8*, and *abi5-7*, only *abi1-1* was slightly stained, whereas the only ABA biosynthetic mutant, *aba1-1*, showed a conspicuously intense level of staining (**Figure 1**). This result implied that *ABA1* knock-out increases the permeability of the seed coat, likely through a disruption in seed coat suberin (Beisson et al., 2007; Vishwanath et al., 2013; Yadav et al., 2014; Gou et al., 2017). However, the deletion of major ABA signaling factors (PYR1, PYL1, PYL2, PYL4, PP2C, and SnRK2.2, 2.3, 2.6) constituting the canonical ABA signaling pathway (Leung et al., 1994; Meyer et al., 1994; Parcy et al., 1994; Gosti et al., 1999; Finkelstein and Lynch, 2000; Merlot et al., 2001; Fujii and Zhu, 2009; Fujita et al., 2009; Ma et al., 2009; Nakashima et al., 2009; Park et al., 2009) resulted in no or weakly significant alterations in seed coat permeability. The increased permeability of the seed coat induced by *ABA1* disruption prompted a further investigation of whether ABA1 is involved in the formation of suberin in seed coats.

### Isolation of *Arabidopsis aba1* knock-out mutant

**Figure 2:**
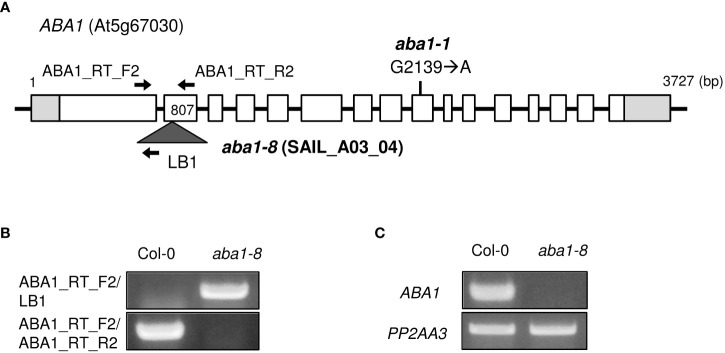
Isolation of T-DNA insertional knock-out mutant *aba1-8*. **(A)** Genomic organization of *ABA1* gene with a point mutation (G2139 → A) in *aba1-1* (Koornneef et al., 1982) and an insertional T-DNA in *aba1-8*. The white boxes indicate the exons, light gray boxes indicate the untranslated region, and the dark gray triangle indicates the inserted T-DNA. Arrows indicate the binding sites of the primers used for T-DNA mutant isolation and confirmation of the knockout mutation. **(B)** T-DNA genotyping of Col-0 and *aba1-8*. ABA1_RT_F2 and ABA1_RT_R2 are *ABA1*-specific primers and LB1 is a T-DNA-specific primer. **(C)** RT-PCR analyses of Col-0 and aba1-8. *PP2AA3* (At1g13320) was used for normalization.

The genomic organization of *aba1-1* and *aba1-8* are described in **Figure 2A**. *aba1-1* is a substitution mutant, in which the 2139^th^ G is substituted with an A, resulting in the early termination of translation (Koornneef et al., 1982; Barrero et al., 2005). To carry out our research on ABA1, we attempted to obtain an additional *aba1* mutant allele. A T-DNA insertional mutant, SAIL_A03_04, was obtained from ABRC, and T-DNA genotyping was performed to verify that a homozygous mutant line had been obtained. PCR analysis with ABA1_RT_F2 and LB1 primers resulted in a PCR band with the *aba1* mutant, but not with the Col-0 wild-type, whereas PCR with a pair of gene-specific primers (ABA1_RT_F2/ABA1_RT_R2) resulted in a PCR band with Col-0 but not with the *aba1* mutant, which confirmed the homozygous T-DNA insertion in the *aba1* mutant (**Figure 2B**). Total RNA was isolated from 9-day-old seedlings of the selected homozygotes and analyzed by performing RT-PCR on *ABA1*, which verified that it was an *aba1* knock-out mutant (**Figure 2C**). RNA quality and quantity were normalized based on the *PP2AA3* gene (At1g13320). The selected *aba1* knock-out mutant was named *aba1-8*, and it was found to harbor its T-DNA insertion in the second exon, as shown in **Figure 2A**.

### *Arabidopsis aba1-1* and *aba1-8* mutants showed retarded growth and abnormal seed development

**Figure 3:**
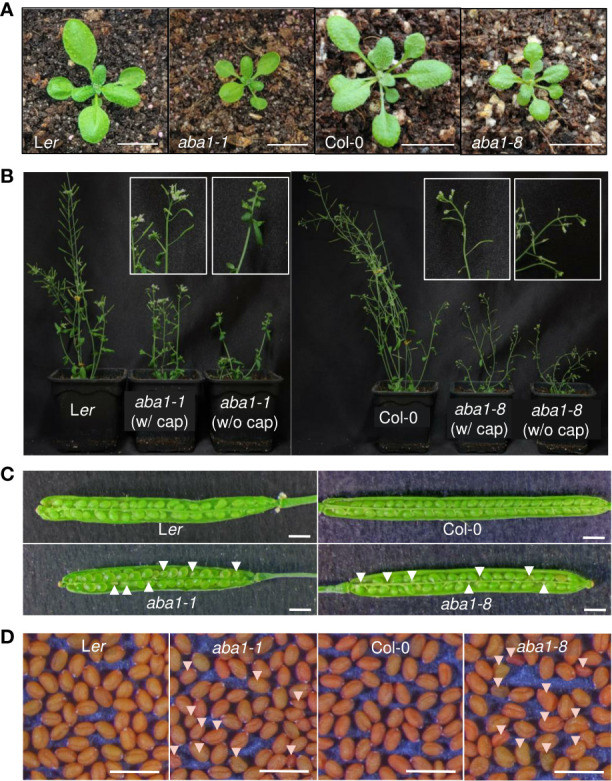
Phenotypes of *aba1-1* and *aba1-8* compared to their corresponding wild-types. **(A)** Three-week-old rosette leaves of L*er*, *aba1-1*, Col-0, and *aba1-8* (from the left). Bar = 1 cm. **(B)** Six-week-old L*er*, *aba1-1*, Col-0, and *aba1-8* (from the left). Since the mutants had difficulty producing siliques on their own, they were grown with transparent canopies made of plastic wrap to assist in fertilization. White boxes in the figure show a 10× magnified shoot of *aba1-1* and *aba1-8* grown with and without the plastic canopy (cap), as a representative. **(C)** Opened developing siliques 8–10 DAF (days after flowering) of L*er*, *aba1-1*, Col-0, and *aba1-8*. White arrows indicate developing seeds with aborted or shrunken phenotypes. Bar = 1 mm. **(D)** Mature seeds of L*er*, *aba1-1*, Col-0, and *aba1-8*. White arrows indicate seeds with an aberrant morphology. Bar = 1 mm.

The morphological and developmental phenotypes of *Arabidopsis aba1-1* and *aba1-8* were then examined. *Arabidopsis* wild-types (Landsberg *erecta* [L*er*] and Columbia-0 [Col-0]) and *aba1* mutants were photographed at 3 and 6 weeks after germination. Both *aba1-1* and *aba1-8* plants showed the significant retardation of growth and reductions in sizes (**Figures 3A, B**). Critically, the *aba1* mutants showed abnormal siliques, since silique development was restrained before its actual appearance or in the midst of its elongation (**Figure 3B**). Then, homozygous *aba1-1* and *aba1-8* mature seeds were obtained through the application of a plastic canopy on top of the plants to assist in fertilization and silique development, by reducing water loss. To investigate if seed development was hindered, developing siliques at 8 to 10 DAF (days after flowering) were opened, and developing seeds were observed under a microscope. The development of some seeds of *aba1-1* and *aba1-8* was found to be aborted or the developing seeds were decreased in size (**Figure 3C**). After harvesting the mature seeds, we observed that some seeds of *aba1-1* and *aba1-8* had an abnormal shape and relatively small or large sizes (**Figure 3D**).

### *Arabidopsis aba1-1* and *aba1-8* mutant seeds showed defective suberin deposition in coats

**Figure 4:**
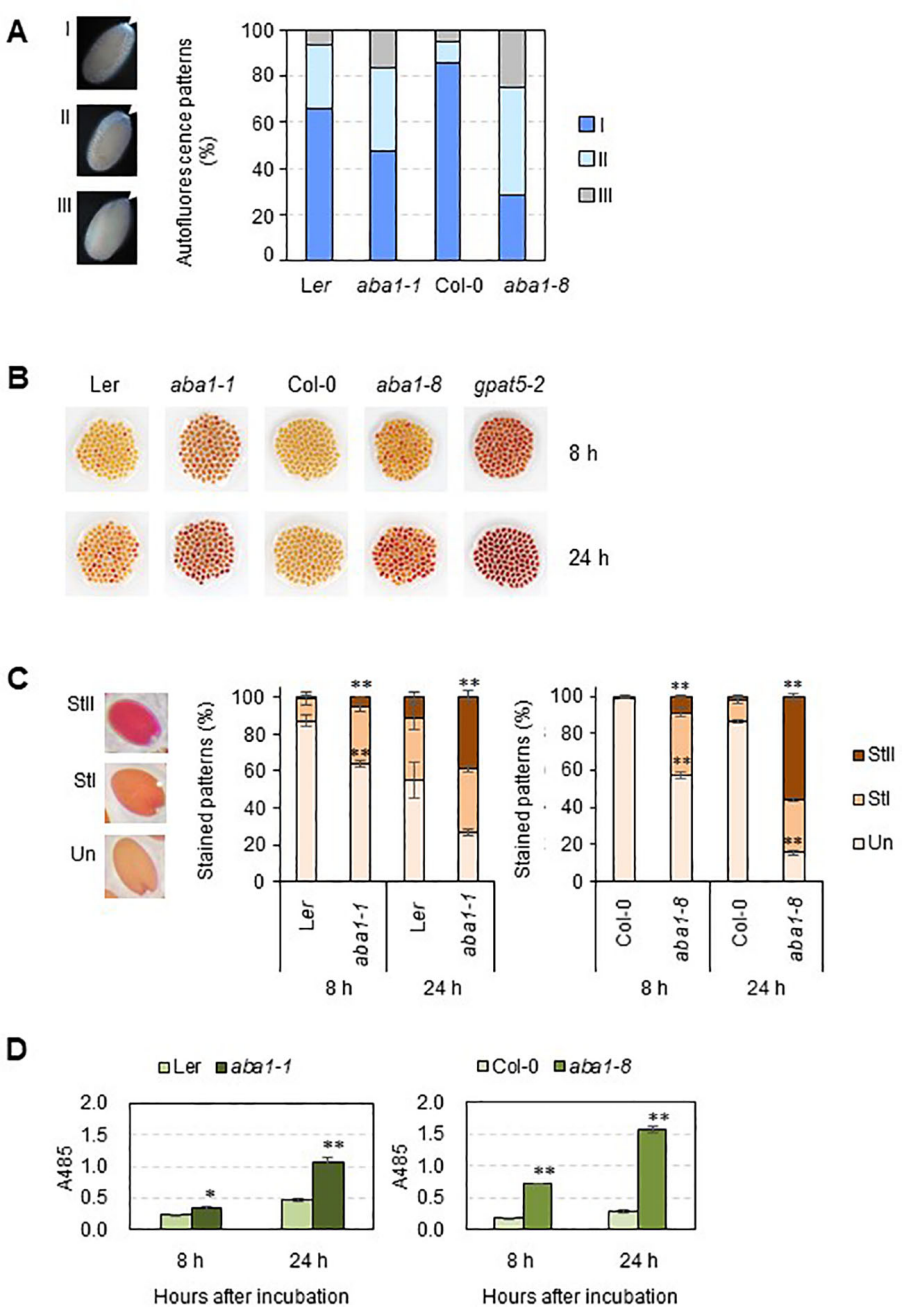
UV autofluorescence observations **(A)** and seedcoat permeability assay **(B–D)** on wild-types (L*er* and Col-0), *aba1-1*, and *aba1-8*. **(A)** Autofluorescence under UV was observed with a fluorescence microscope. Arrows indicate autofluorescence in the chalazal region of the seed coat. Seeds were divided into three groups (I, strong; II, weak; III, none or almost none) based on their autofluorescence intensity, and one seed from each group is shown as a representative. Percentages of seeds belonging to each group, I, II, and III, were determined and are shown on the right. Values were obtained from 100–110 total seeds. **(B)** WT and *aba1* mutants were immersed in 1% (w/v) tetrazolium salt solution for 8 and 24 h. After the incubation, seeds were photographed with a digital camera. **(C)** Stained seeds were divided into three groups based on the degree of staining (Un, unstained; StI, weakly stained; StII, strongly stained). Percentages of the seeds belonging to each group were determined. Each value is an average of three replicates with 100–105 seeds per replicate. Error bars indicate ± SE of the average. Asterisks indicate the significance of statistical differences compared to the WT, determined using a Student’s t-test. (*p < 0.05, **p < 0.01). **(D)** Amount of formazan produced in response to the penetration of tetrazolium salt into the embryos, as measured based on the absorbance at 485 nm at time points of 8 and 24 hours after incubation. Asterisks indicate the significance of statistical differences compared to the WT, determined using a Student’s t-test. (*p < 0.05, **p < 0.01).

Because phenolic compounds, such as ferulates, are the components of suberin, suberin is detectable based on its autofluorescence under ultraviolet light (Lee and Suh, 2018). Approximately 100 seeds of the wild-types, *aba1-1*, and *aba1-8* were observed under UV light with a fluorescence microscope. The seeds were sorted into three groups (I, strong; II, weak; and III, none or almost none) based on their autofluorescence signal intensity under UV light. The *aba1* mutants had a significantly lower abundance of suberin polyesters accumulated in their seed coats. Moreover, *aba1-1* had approximately 18% more seeds that showed no or almost no UV autofluorescence signals (group III) compared to wild-type numbers, and *aba1-8* had approximately 58% more seeds belonging to group III compared to numbers in the wild-type (**Figure 4A**). A TZ permeability assay was performed with both *aba1* allele-mutant and wild-type seeds, and the seeds were observed after 8 and 24 h from the onset of staining. TZ-tested seeds were divided into three different groups, namely unstained (Un), weakly stained (StI), and intensely stained (StII) according to the staining intensity. *Arabidopsis aba1-1* and *aba1-8* had approximately 28% and 54% more intensely stained seeds (group StII) than their corresponding wild-types at 24 h of staining, respectively (**Figures 4B, C**). This was further confirmed by measuring the amount of formazan produced *via* the reduction of TZ salt that permeated into the embryos. *aba1-1* seeds produced approximately 1.5- and 2-fold more formazan than L*er* seeds at 8 and 24 h, respectively. And, *aba1-8* seeds produced approximately 4- and 5.6-fold more formazan than Col-0 seeds at 8 and 24 h, respectively (**Figure 4D**).

### Suberin monomer amounts and composition were altered in *Arabidopsis aba1-1* and *aba1-8* seed coats

**Figure 5:**
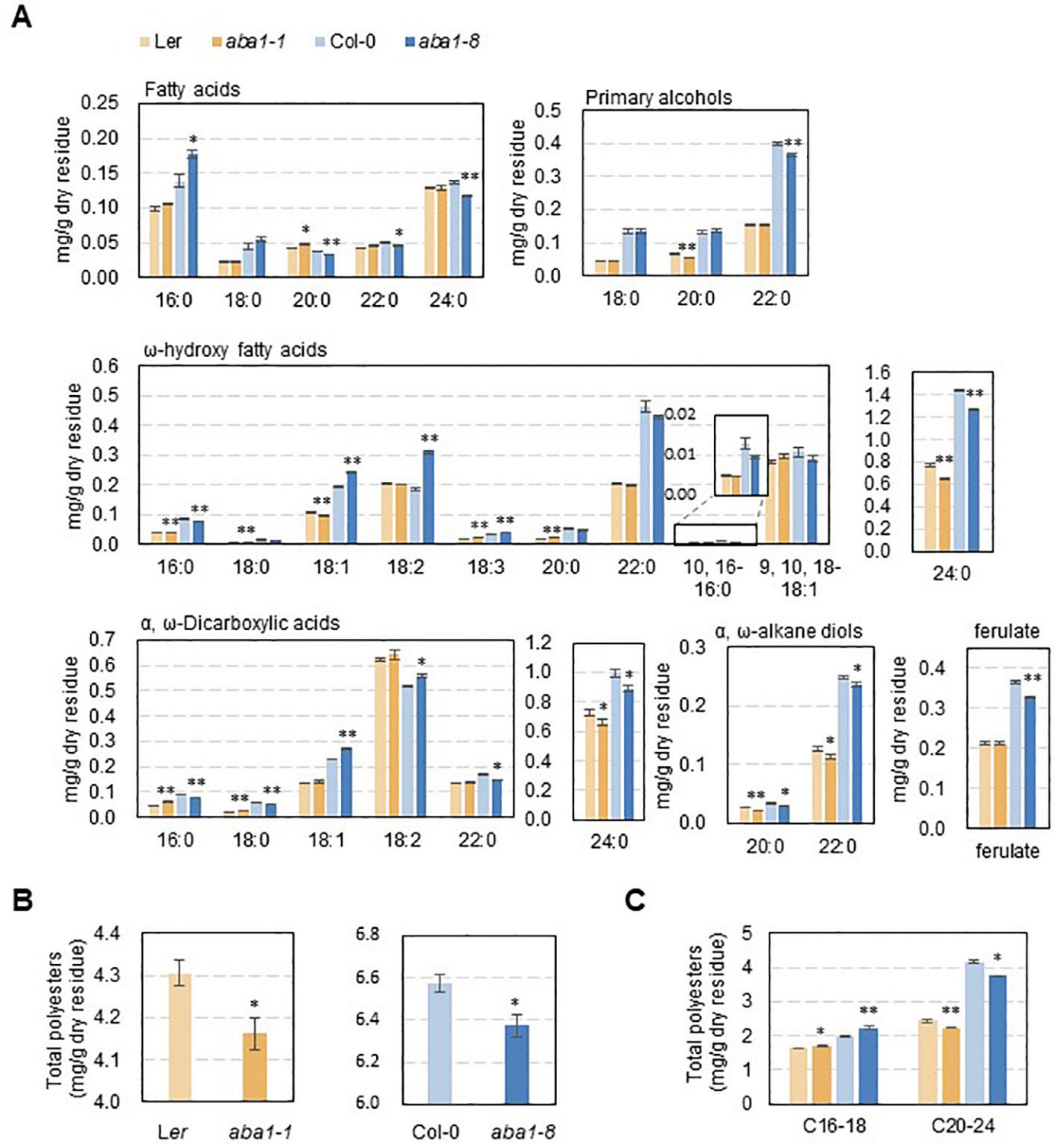
Suberin analysis in wild-type (L*er* and Col-0), *aba1-1*, and *aba1-8* seeds. Seed coat polyester monomers **(A)** and total polyester quantity **(B, C)** of L*er*, *aba1-1*, Col-0, and *aba1-8*. The seed dry residue was obtained after grinding and gradual delipidation with chloroform and methanol. Suberin monomers were released from the dry residues by depolymerization through a base catalysis method and analyzed *via* GC-FID. Error bars indicate ± SE of the average of three to five independent experiments. Asterisks indicate the statistical differences compared to the wild-type, in which *p < 0.05, **p < 0.01.

To examine whether suberin monomer amounts and composition were altered in *aba1* seed coats, suberin monomer analysis was performed. Suberin polyesters were extracted from the seed coats of wild-types (Col-0 and L*er*) and *aba1* mutants and hydrolyzed, and then, suberin monomers were analyzed through gas chromatography with a flame ionization detector (GC-FID). Interestingly, the levels of C24:0 ω-hydroxy fatty acids and C24:0 α, ω-dicarboxylic acids, which account for the majority of seed coat suberin monomers, were decreased by approximately 16% and 10% in *aba1-1*, respectively, compared with levels in L*er* and by approximately 12% and 10% in *aba1-8*, respectively, relative to levels in Col-0. The levels of C20:0 and C22:0 α,ω-alkane diols were also decreased by approximately 22% and 15% in *aba1-1*, respectively, relative to those in L*er* and by approximately 15% and 6%, respectively, in *aba1-8* compared to those in Col-0. The amount of ferulate decreased by approximately 11% in only *aba1-8* relative to Col-0 (**Figure 5A**). Total suberin monomer amounts were decreased by approximately 3% in both *aba1-1* and *aba1-8* mutants compared to those in their corresponding wild-types (**Figure 5B**). Amounts of suberin monomers were also analyzed by totaling the levels of LC (C16-18) and VLC (C20-24) monomers. Amounts of VLC monomers were decreased by approximately 9% and 10% in *aba1-1* and *aba1-8* seed coats, respectively, compared to those in their wild-types, whereas amounts of LC monomers were increased by approximately 4% and 12% in *aba1-1* and *aba1-8* seed coats, respectively, compared to those in their corresponding wild-types (**Figure 5C**).

### Developing siliques of *Arabidopsis aba1-1* and *aba1-8* showed decreased expression of genes involved in suberin formation

**Figure 6:**
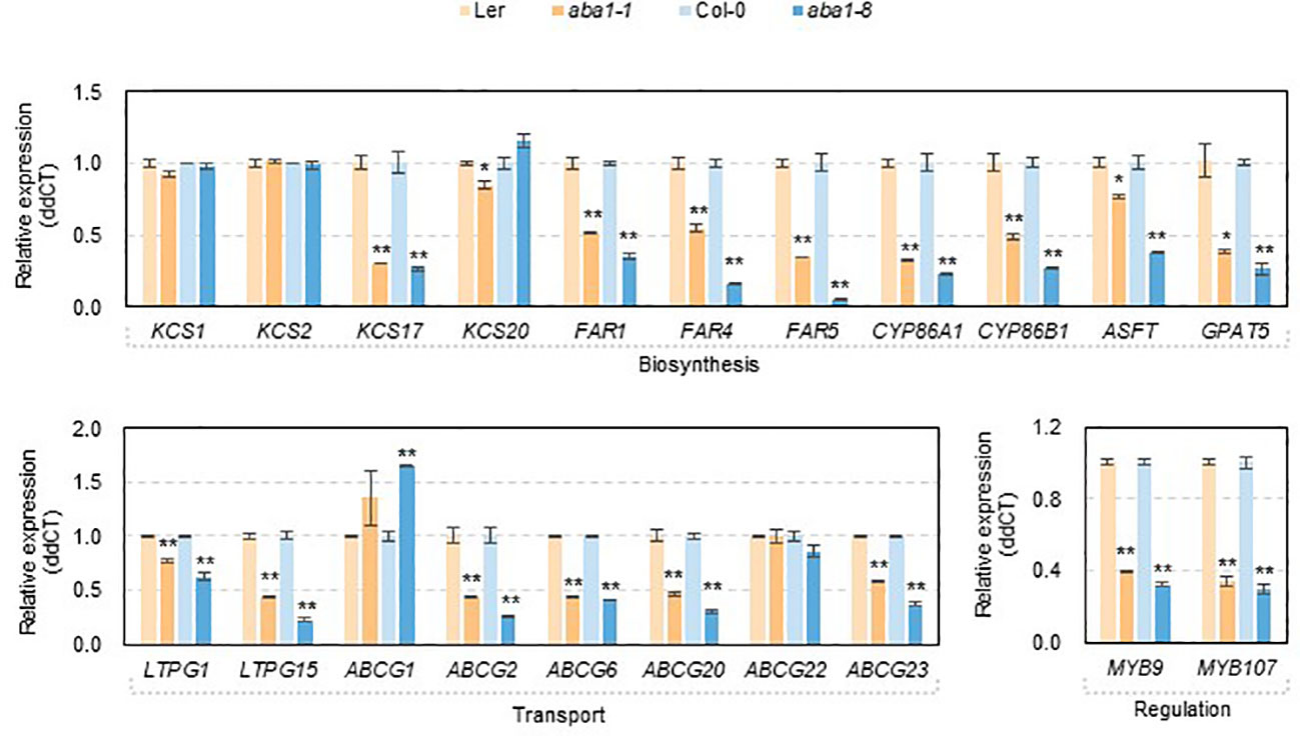
Expression of genes involved in suberin formation in developing siliques of wild-types (L*er* and Col-0), *aba1-1*, and *aba1-8*. The expression of genes involved in suberin formation in developing siliques (4–10 days after flowering; DAF) in wild-types (L*er* and Col-0) and *aba1* mutants was analyzed by performing quantitative PCR. The transcription levels were normalized based on the expression level of *ACTIN7* (At5g09810), and the raw data were analyzed using the ddCT method. The error bars indicate ± SE of three replicates. A Student’s t-test was used to determine the significance, which is denoted with asterisks (*p<0.05, **p<0.01).

To investigate if changes in the amounts and composition of suberin monomers were related to the transcript levels of genes involved in suberin formation, RT-qPCR was carried out. Because *aba1* seeds were very sensitive to atmospheric conditions (approximately 30 to 40% RH) during the dissection of developing seeds and silique walls, total RNA was isolated from developing siliques at 4–10 DAF, converted to cDNA, and used for the RT-qPCR analysis. *KCS1*, *KCS2*, *KCS17*, *KCS20*, *FAR1*, *FAR4*, *FAR5*, *CYP86A1*, *CYP86B1*, *GPAT5*, *ASFT*, *LTPG1*, *LTPG15*, *ABCG1*, *ABCG2*, *ABCG6*, *ABCG20*, *ABCG22*, *ABCG23*, *MYB9*, and *MYB107* genes were selected for RT-qPCR analysis based on seed coat-specific or seed coat-preferential expression, based on previous reports or the eFP browser (http://bar.utoronto.ca) (**Figure S2**) (Beisson et al., 2007; Compagnon et al., 2009; Lee et al., 2009; Molina et al., 2009; Lashbrooke et al., 2016; Gou et al., 2017; Lee and Suh, 2018). Among four β-ketoacyl CoA synthases (KCSs), a dramatic decrease in the transcript levels of *KCS17* was observed in *aba1-1* and *aba1-8*, compared with levels in each wild-type. The expression levels of three genes encoding fatty acyl-CoA reductases, *FAR1*, *FAR4*, and *FAR5*, and two fatty acid ω-hydroxylases, *CYP86A1* and *CYP86B1*, were all dramatically decreased in the mutants compared to those in the wild-types. Two types of acyltransferases, aliphatic suberin feruloyl transferase (*ASFT*) and glycerol-3-phosphate acyltransferase 5 (*GPAT5*), also showed decreased transcript levels in *aba1-1* and *aba1-8* compared to wild-type levels, whereas the change in the expression level of *ASFT* in *aba1-1* was not as dramatic as that in *aba1-8*. In terms of the expression of genes involved in the transport of suberin monomers, expression levels of two *LTPG*s and six *ABCG*s, *LTPG1*, *LTPG15*, *ABCG2*, *ABCG6*, *ABCG20*, and *ABCG23* were significantly downregulated in *aba1-1* and *aba1-8* compared to levels in their corresponding wild-types. The transcript levels encoding the MYB transcription factors MYB9 and MYB107, which are known to regulate suberin biosynthetic genes mainly in seed coats, were decreased significantly in *aba1-1* and *aba1-8* relative to levels in their corresponding wild-types (**Figure 6**).

### *Arabidopsis aba1-1* and *aba1-8* seeds showed defective mucilage, but no alterations in proanthocyanidin deposition

Because the disruption of *ABA1* caused the defective formation of seed coat suberin, we attempted to observe changes in mucilage and proanthocyanidin deposition in *aba1* seed coats. Seeds were stained with 0.01% (w/v) ruthenium red and 1% (w/v) vanillin to visualize mucilage and proanthocyanidin, respectively. The lack of ABA caused a decrease in the thickness of mucilage (**Figure S3A**), but no noticeable changes in the accumulation of proanthocyanidins, based on mutants of both alleles of *aba1* in seeds, relative to those observed in the wild-types (**Figure S3B**).

## Discussion

ABA is a major phytohormone that regulates seed development. Through an intricate signaling network, it is involved in de-greening and obtaining desiccation tolerance in seeds, as well as in the accumulation of seed storage products, which leads to the maturation and dormancy of the seeds (Ali et al., 2022). In this process, the hydrophobic seed coat polyester is known to play a critical role. Through its osmotic sealing of the seed, seed coat polyesters maintain the impermeability of the seed coat to exclude oxygen, water, heavy metals, and nutrients (Beisson et al., 2007; Fedi et al., 2017; de Silva et al., 2021), which protects the seed itself and maintains seed dormancy. A defective seed coat suberin layer causes increased sensitivity to germination inhibiting factors such as NaCl, paclobutrazol, and ABA, and reduced dormancy at low temperatures (Fedi et al., 2017). Given the relevance of this deep connection between the role of ABA in seed development and the contribution of seed coat polyesters to achieving this role, it could be inevitable that ABA regulates the biosynthesis of seed coat polyesters. In this study, we revealed that ABA is involved in seed coat suberization based on the following evidence: (i) decreased UV autofluorescence signals in the chalazal regions of *aba1-1* and *aba1-8* seeds, (ii) increased permeability of *aba1* seed coats to TZ salts, (iii) significantly decreased levels of suberin monomers, especially C24 α,ω-dicarboxylic acids and C24 ω-hydroxy fatty acids, in *aba1* seed coats relative to those in the wild-type, and (iv) decreased expression levels of several genes involved in suberin accumulation and its regulation.

Even though the *aba1* mutants showed remarkably increased permeability to TZ salts (**Figures 1**, **4B–D**), the actual amount of suberin monomers was not decreased to the extent that was expected (**Figure 5**). It was suggested that a decreased amount of suberin monomers is not always correlated with increased permeability of the suberin layers. Rather, the aliphatic monomer arrangement in the suberin microstructure could contribute to the permeability of suberin (Ranathunge and Schreiber, 2011). Although the actual amount of total suberin monomers decreased only by approximately 3% in *aba1* mutants, this decrease was mainly caused by the noticeable decrease in the amounts of C24 α,ω-dicarboxylic acids and ω-hydroxy fatty acids. The alterations in the suberin aliphatic and aromatic composition might have resulted in changes to the suberin microstructure, which led to increased permeability. Similar results were observed in a study by Lee and Suh (2018), where only approximately 8% of fatty acids (C20–24), primary alcohols (C20–22), ω-hydroxy fatty acids (C22–24) and α,ω-alkanediols (C20–22) were decreased, and no significant differences in total polyester amounts were observed in *ltpg15* seed coats. These slight changes in polyester composition were followed by increased permeability of the *ltpg15* seed coat.

The expression of several *KCS* isoforms was analyzed, as shown in **Figure 6**. Among these, *KCS17* showed a conspicuous decrease in expression in *aba1-1* and *aba1-8* compared to levels in their corresponding wild-types, whereas other *KCS*s did not show any decrease (**Figure 6**). *KCS17* was shown to be expressed predominantly in the seed coat (https://bar.utoronto.ca), suggesting that it can be involved in the synthesis of VLCFAs that are required for the formation of seed coat suberins. Downregulated expression of *KCS17* is consistent with the decreased amount of total VLC monomers (C20 to C24) along with the increased amount of total long-chain monomers (C16 and C18) in the seed coats of *aba1-1* and *aba1-8* (**Figure 5C**). *FAR1*, *FAR4*, and *FAR5* are involved in the synthesis of C22, C20, and C18 primary alcohols, respectively, which comprise root waxes and suberin polyesters of the root and seed coat in *Arabidopsis* (Domergue et al., 2010; Vishwanath et al., 2013). *FAR*s showed severely decreased expression in developing siliques of *aba1-1* and *aba1-8* (**Figure 6**). This reduced expression is consistent with decreased levels of C20 and C22 primary alcohols in the seed coat polyesters of *aba1-1* and *aba1-8*, respectively. CYP86B1 catalyzes the ω-hydroxylation of VLC fatty acyl-CoAs to ω-hydroxy acids of C22 or C24. This was reflected by the decreased amounts of C24 ω-hydroxy acids in *aba1-1* and *aba1-8* compared to those in their respective wild-types. Decreased levels of VLC monomers were also observed in suberin layers of *ltpg15* seed coats (Lee and Suh, 2018), which aligned with the decreased expression of *LTPG15* shown in **Figure 6**.

Data from the eFP browser database (https://bar.utoronto.ca) showed that the expression of *MYB107*, *MYB9*, *FAR4*, *FAR5*, *CYP86B1*, *GPAT5*, *ASFT*, *LTPG15*, *ABCG6*, *ABCG20*, and *ABCG23* was induced in *Arabidopsis* seedlings after 3 h of 10 µM ABA treatment (**Figure S4**). Exogenous ABA treatment was also found to induce expression of the fluorescent protein-encoding gene *mCITRINE-SYP122* in *Arabidopsis* roots under the control of the *GPAT5* promoter (Barberon et al., 2016; Shukla et al., 2021; Leal et al., 2022). In kiwifruit, the expression of *AchnMYB107*, which is a homologue of *AtMYB107*, is increased in response to exogenous ABA treatment, followed by the induction of *AchnCYP86A1* and *AchnFAR* expression, which are speculated to encode a fatty acid ω-hydroxylase and fatty acyl reductase, respectively, responsible for wound suberization in kiwifruit (Wei et al., 2020a; Wei et al., 2020b). The previous reports, along with the data shown in **Figure 6** showing that the expression of several suberization-related genes was decreased in an ABA biosynthetic mutant, suggest that ABA regulates suberization *via* the direct or indirect induction of several genes involved in suberization-related genes.

Decreased expression of suberization-related genes in *aba1* mutants can be considered based on transcriptional regulation *via* transcription factors. MYB9 and MYB107 were reported to be transcription factors that positively regulate the expression of suberin biosynthetic genes in *Arabidopsis* seed coats (Lashbrooke et al., 2016; Gou et al., 2017). *MYB107* was found to be predominantly expressed in developing *Arabidopsis* siliques, and its disruption severely increased seed coat permeability to TZ salts. Most suberin monomer components were decreased in *myb107* seed coats, and in particular, an approximate 50% decrease in the levels of C24 α,ω-dicarboxylic acids and C24 ω-hydroxy fatty acids were most prominent. The levels of *FAR1*, *FAR4*, *FAR5*, *CYP86A1*, *CYP86B1*, *GPAT5*, *ASFT*, and *LTPG15* were significantly downregulated, but no alterations were observed in the levels of *LTPG1* and *ABCG2*, *6*, *20*, and *23* transcripts in developing *myb107* seeds. In addition, the disruption of *Arabidopsis MYB9* causes decreased expression of *CYP86A1* in developing seeds (Lashbrooke et al., 2016; Gou et al., 2017). In this study, the expression of *MYB9* and *MYB107*, together with *KCS17*, *FAR1*, *FAR4*, *FAR5*, *CYP86A1*, *CYP86B1*, *GPAT5*, *ASFT*, and *LTPG15*, was severely downregulated in developing *aba1-1* and *aba1-8* siliques (**Figure 6**). Taken together, ABA-induced MYB9 and MYB107 are upstream factors involved in the expression of *FAR1*, *FAR4*, *FAR5*, *CYP86A1*, *CYP86B1*, *GPAT5*, *ASFT*, and *LTPG15* in *Arabidopsis* seed coats. However, the transcriptional factors required for the ABA-dependent expression of *ABCG2*, *6*, *20*, and *23* remain to be further investigated.

Interestingly, ABA is likely to regulate the synthesis of seed coat suberins *via* a seed coat-specific signaling pathway, which has not yet been revealed. The knock-out of *ABA1* resulted in defective suberin layers (**Figure 1**, **4**, **5**), but the disruption of ABA signaling factors, specifically *SnRK2*, *ABI3*, *ABI5*, and *PYR*/*PYL*/*RCAR*, resulted in no remarkable alterations in seed coat permeability to TZ salt (**Figure 1**). When ABA-deficient *aba3* and ABA-insensitive mutants *112458*, *abi1-1*, *snrk2.236*, and *abf234* were tested for leaf cuticle permeability to toluidine blue O, *abf234*, an ABA signaling triple mutant with mutations in ABA-responsive element binding factors 2, 3 and 4, was not affected in terms of leaf cuticle permeability, unlike that observed in the other ABA-deficient or ABA-insensitive mutants (Cui et al., 2016). This result suggests that the canonical ABA signaling pathway that responds to SnRK2 kinases is involved in cuticle formation but that there are different downstream ABA-responsive factors involved in the process (Cui et al., 2016). Likewise, another pathway involved in ABA signaling functions in the seed coats, related to ABA in this case, could be suggested. *abi1-1*, which also displayed increased seed coat permeability (**Figure 1**), is a dominant negative mutant that has a defective PP2C, continuously sequestering SnRK2 regardless of the presence of ABA. Hence, the canonical ABA signaling factor ABI1 is still considered to be involved in ABA signaling in seed coats. In addition, the ABA receptors PYL3, 6, 8, and 9 could be candidates that transmit the signals involved in seed coat suberization, as they are highly and predominantly expressed in the seed coat or seed coat chalazal region (**Figure S5**).

**Figure 7:**
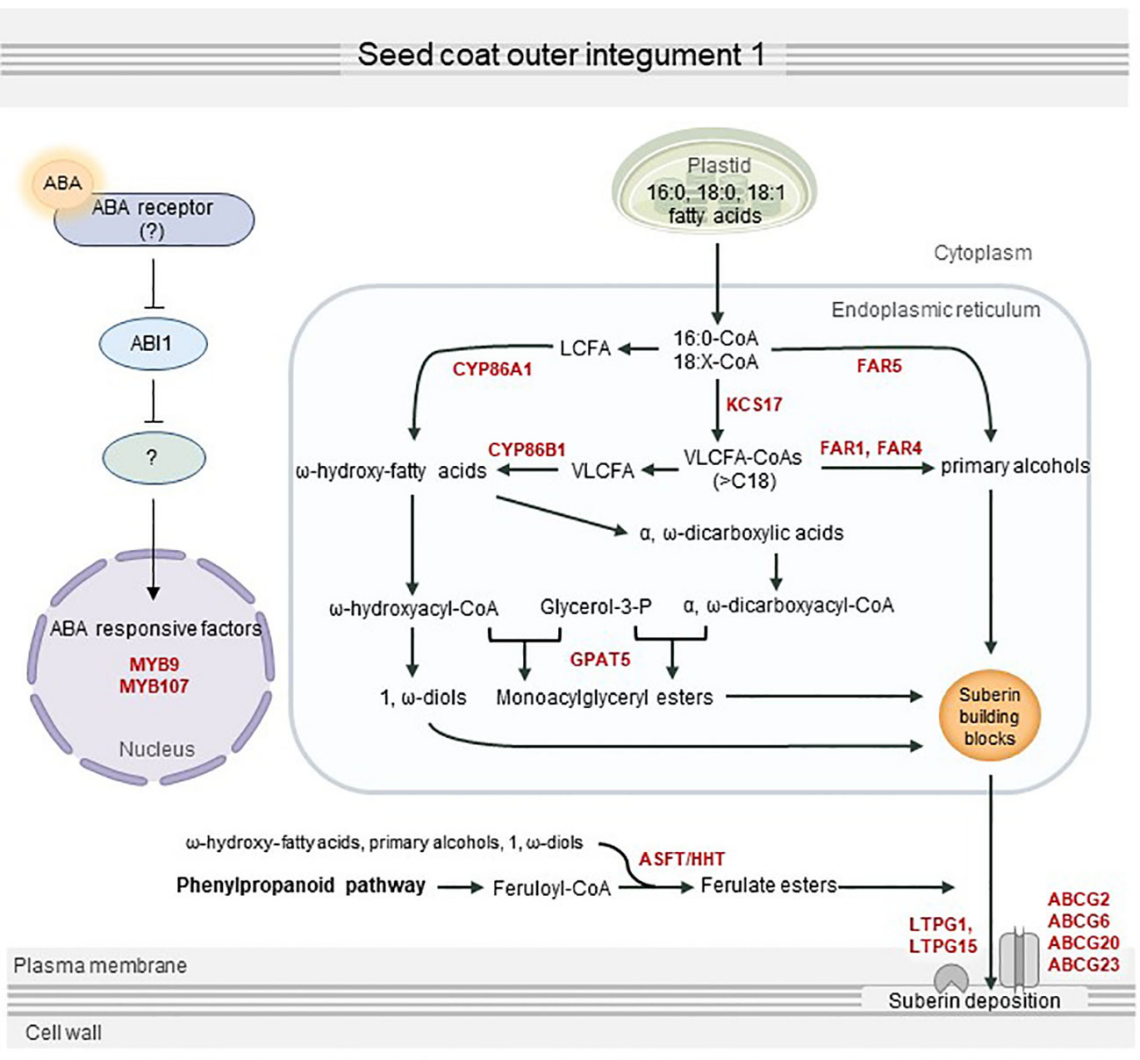
Proposed overview of seed coat suberization regulated by abscisic acid (ABA). ABA transferred to the seed coat is perceived by an unknown ABA receptor, which transmits an ABA signal *via* the sequestration of ABI1 phosphatase protein 2C. MYB107, MYB9, and other suberization-related transcription factors are directly or indirectly activated by ABA signaling, which in turn upregulates suberin biosynthesis and transport genes, including *KCS17*, *FAR1*, *FAR4*, *FAR5*, *CYP86A1*, *CYP86B1*, *GPAT5*, *ASFT*, *ABCG2*, *ABCG6*, *ABCG20*, *ABCG23*, *LTPG1*, and *LTPG15*. This contributes to the proper development of the seed by stimulating suberin deposition in the seed coat.

In summary, we revealed that ABA transmits downstream signals *via* a partially preserved canonical ABA signaling pathway, which eventually activates seed coat suberization through positive transcription factors including MYB107 and MYB9. This in turn can upregulate the expression of several suberization-related genes, resulting in seed coat suberin deposition (**Figure 7**). This study provides a better understanding of the process of seed coat suberization and a basis to investigate an unknown ABA signaling pathway functioning in the seed coat.

## Data availability statement

The original contributions presented in the study are included in the article/**Supplementary Material**. Further inquiries can be directed to the corresponding author.

## Author contributions

MS designed the research. JC and HK performed the experiments and analyzed the data with MS. JC and MS wrote the paper. All authors contributed to the article and approved the submitted version.
