# Supplementary material for: Disruption of the ABA1 encoding zeaxanthin epoxidase caused defective suberin layers in Arabidopsis seed coats
Source: Front Plant Sci. 2023 Mar 15;14:1156356. doi: 10.3389/fpls.2023.1156356 (PMC10050373; doi:10.3389/fpls.2023.1156356)
Supplement: Supplementary file 1 [file DataSheet_1.pdf]

Supplementary table 1. List of primers used in this study

Experiment	Primer name	Sequence (5’-3’)
Genotyping of *QC3*, *QL3*, and *snrk2.2/3/6+*	PYR1_F1	GTG GAT TAC CGG CGA ACA C
	PYR1_R1	CAC GTC ACC TGA GAA CCA C
	PYL1_F1	GAC GAA TTC ACC CAA CTC TCC
	PYL1_R1	GTT CCG GTT CAT AGC TTC AGT G
	PYL2_F1	GAA AGG CCT AAC CGA TGA AGA GC
	PYL2_R1	GGC AAC TCC GAG TTT CTG AAG G
	PYL4_F1	CGT TCA CCG TCC TTC TTC C
	PYL4_R1	GCT CTC AGC CGC AGT ATT CTC
	SnRK2.2_F1	GGT GAT TTT GAC GCC TTC CC
	SnRK2.2_R1	GCT CCT GTG GAT CCT CGA ATG
	SnRK2.3_F1	CAT TTT GAC GCC GAC TCA TCT G
	SnRK2.3_R1	GAA CGG ATA CGC TCC AAC C
	SnRK2.6_F1	GGA TCG ACC AGC AGT GAG TG
	SnRK2.6_R1	CTG CAT TGC AGA TTC GCT CG
	LBb1.3	ATT TTG CCG ATT TCG GAA C
	GK_LB_F1	ATA TTG ACC ATC ATA CTC ATT GC
	SAIL_LB1	GCC TTT TCA GAA ATG GAT AAA TAG CCT TGC
Isolation of *aba1* T-DNA knockout mutant	ABA1_RT_F2	GGC CCG ATT CAA ATA CAG AGC A
	ABA1_RT_R2	CCA ACC GCA CGT GCT AGA ATC
	LB1	GCC TTT TCA GAA ATG GAT AAA TAG CCT TGC
	At1g13320_RT_F1	CAG CTC AGC TCC TGA TTC ACT ACC
	At1g13320_real_R	GAA CCA AAC ACA ATT CGT TGC TG
Quantitative real-time PCR	MYB9-qRT-F1	CTC TCA CAT TCT TGA GGA TGA G
	MYB9-qRT-R1	TGA AAA CCG GGT TGT ATG GG
	MYB107-qRT-F1	GGA AAC TTC ACA GCT GAG GA
	MYB107-qRT-R1	CGT TTA GAT GGT CGG TTC TTG
	KCS1-real-F1	TTC GAG TAG CTC GCT TTG GTA
	KCS1-real-R1	GTT CAA CCA ATC ATT GCA CAA CT
	KCS2_real_F2	CAA CCT CGC TTT CCA ACA AA
	KCS2_real_R2	TCC GGT TTT CTC AAG CAC TG
	KCS6-real-F1	AAG AAG GAC ACG TTG GCA TCA
	KCS6-real-R1	CGA TTA GGG ACG TGA GGA AG
	KCS17-real-F1	TGG CAG ATT GCT TTT GGT AGC
	KCS17-real-R1	GGT ACA TGG AAA CCA ACC AAC
	KCS20_real_F	CAG ATG CTT CAG GTG CAA CC
	KCS20_real_R	TGA ACG GCT GCG ATC AGA
	FAR1-qRT-F1	CAG CTC ATT CGG GAG ACA C
	FAR1-qRT-R1	ACG AGC CGT GAA ATC GTG A
	FAR4-qRT-F1	GCA AGA TTA AGC TGG CGA TGC
	FAR4-qRT-R1	GCC GGG AAT ATG AAT GGT CGT C
	FAR5-qRT-F1	GAG AGA CAA GAA CCG CAA AAT CGA G
	FAR5-qRT-R1	CGT GTG TGA CGA GTC CAG AA
	CYP86A1qRT-F1	GCT CGT TTA CCT CAA GGC TGC T
	CYP86A1qRT-R1	CGG ACG GAA CTC GAG ACA ATC
	CYP86B1_real_F1	TGA CCA TGG AAA TGC AGA GAA G
	CYP86B1_real_R1	TTA GCC ACC TCT CTG GCC TA
	HHT-ASFT-qRT-F1	TGA GAT CCC TCC TGT TAC CGC
	HHT-ASFT-qRT-R1	CCA TAG CTC CAA TTC CAT CGA ACA TAC AGT
	GPAT5_real_F1	CAC TTT CGA GCC TTG TCT CC
	GPAT5_real_R1	CGA AAG AAA GAA ACT GCA AAC AG
	LTPG1 real F1	TCC CAA CTT CTT GTC AGC TCC A
	LTPG1 real R1	CCC TTA TCC GTG GAC GTA GCT G
	AT2g48130_LTP_type5-qRT-F1	CTC CCC TCA CAC AAT GCA AC
	AT2g48130_LTP_type5-qRT-R1	TAC CAA CGG AGG GAA CTG TC
	ABCG1-real F1	GGT AAC GAT ATC GAG CAC GAC TTG

	ABCG1-real R1	CAC CTC CGC TTG TTC TTG CT
	ABCG2-qRT-F1	ACG AGA TGT TTC GCC AGA GGA G
	ABCG2-qRT-R1	ATA TCA ATC CCC GTC GTC ACA CAA G
	ABCG6-real-F1	GCA ACA AGG CGT TAC GGA TCT
	ABCG6-real-R1	CAA GTT TCA AAC CAG TCC TCC A
	ABCG20-qRT-F1	AAT CAG CCT CCT GAA GAG CAT GAG
	ABCG20-qRT-R1	GTT CTT GCT TCC GAT CAA GAG TGT G
	ABCG22-real-F1	TCT TCG GAT CAG CCC ATA TCC
	ABCG22-real-R1	GTT GAT GGA CAC AGC GAA GTC
	ABCG23-real-F1	TTA CCC GCT GGA GTC TAT GG
	ABCG23-real-R1	CCC CAG CAG AGG ATT CGA TAA
