# Supplementary figures and images for: Disruption of the ABA1 encoding zeaxanthin epoxidase caused defective suberin layers in Arabidopsis seed coats
Source: Front Plant Sci. 2023 Mar 15;14:1156356. doi: 10.3389/fpls.2023.1156356 (PMC10050373; doi:10.3389/fpls.2023.1156356)

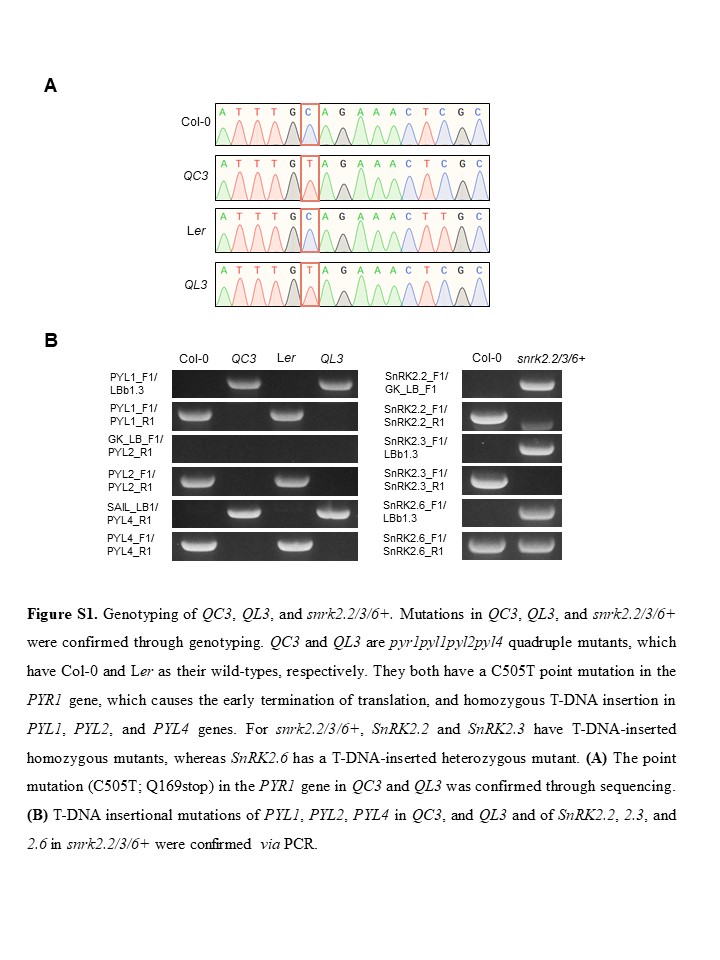

Supplement: Supplementary file 2 [file Image_1.jpeg]

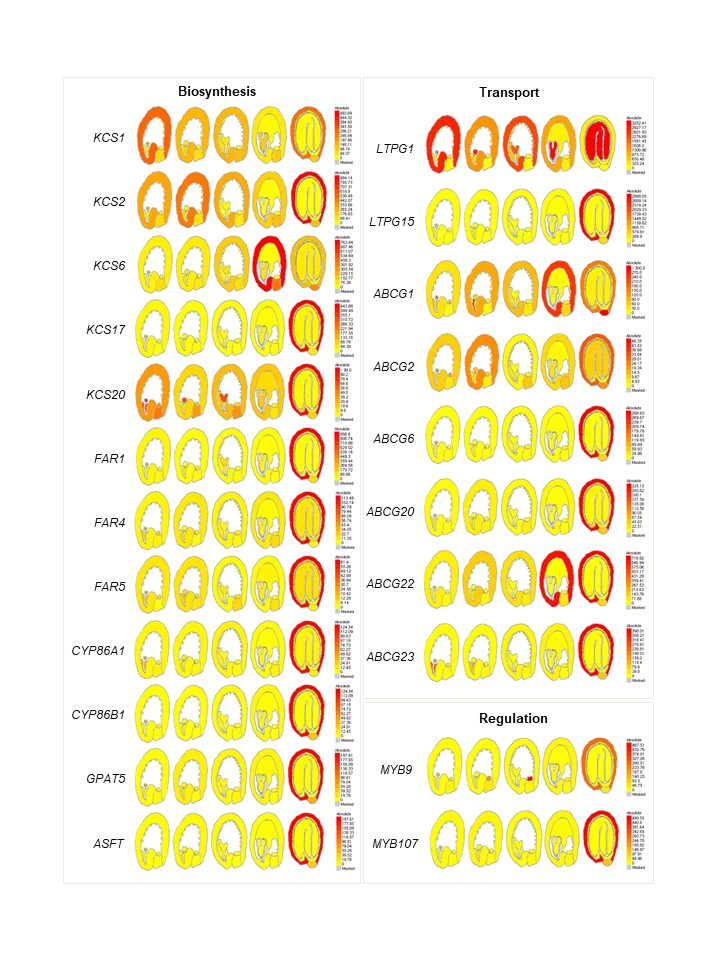

Supplement: Supplementary file 3 [file Image_2.jpeg]

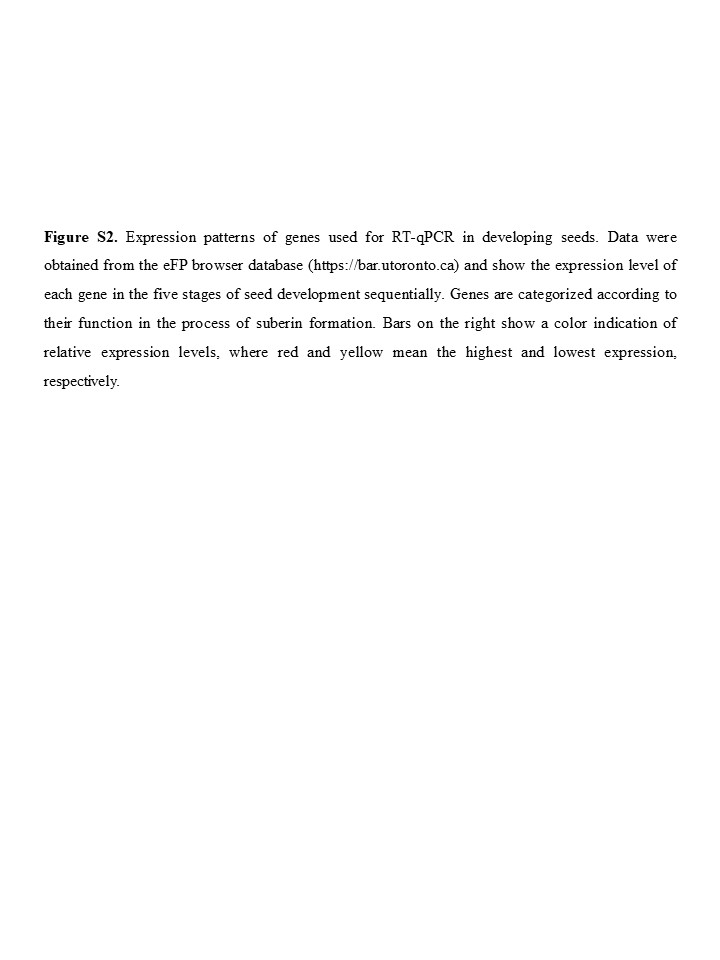

Supplement: Supplementary file 4 [file Image_3.jpeg]

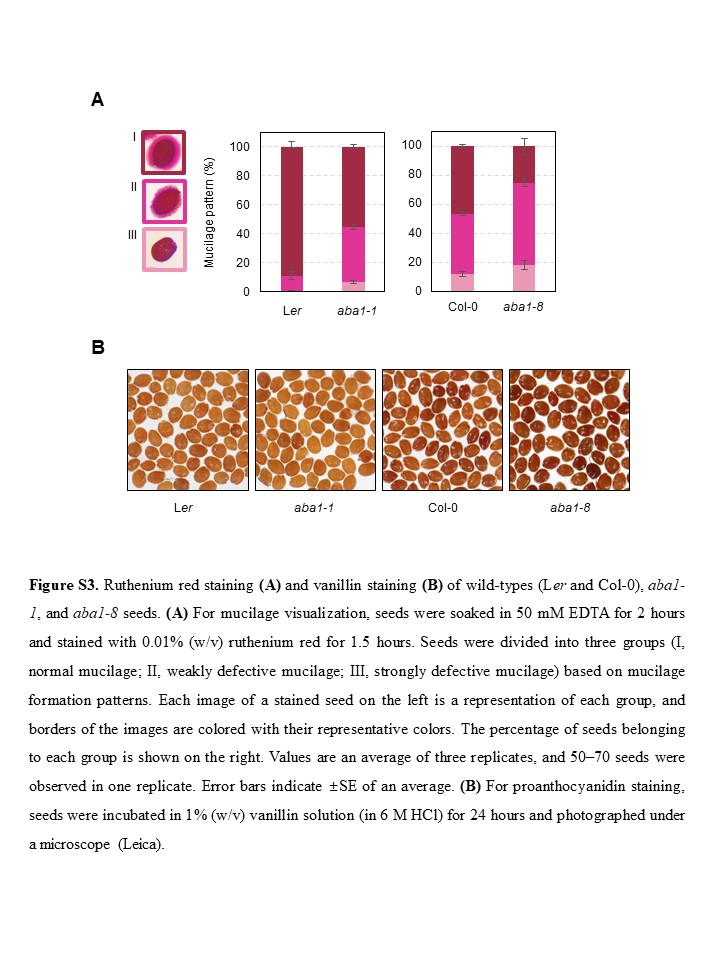

Supplement: Supplementary file 5 [file Image_4.jpeg]

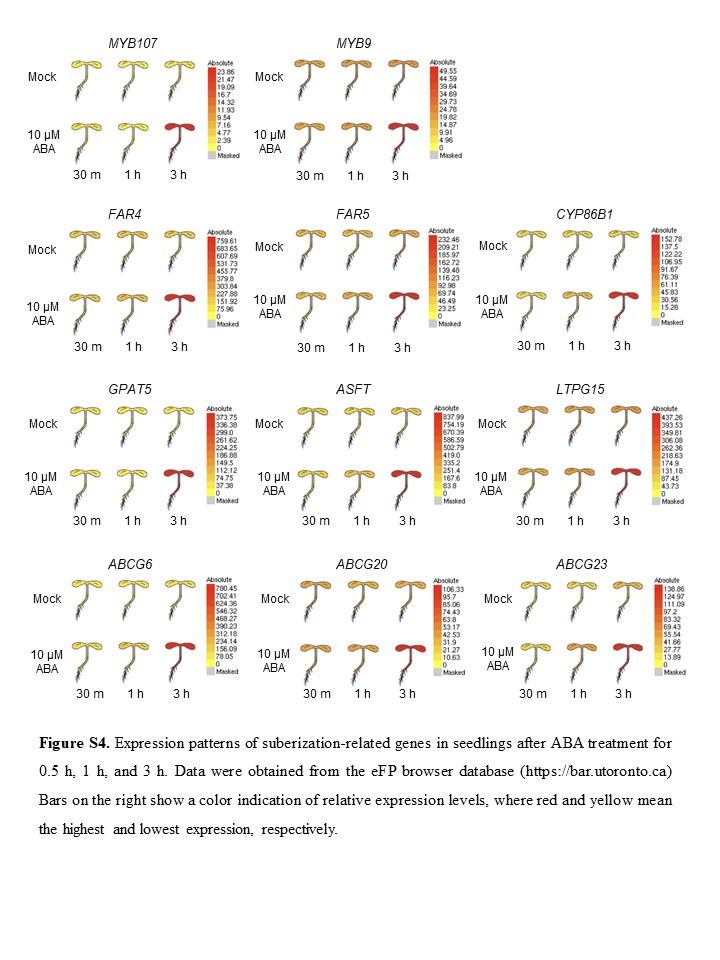

Supplement: Supplementary file 6 [file Image_5.jpeg]

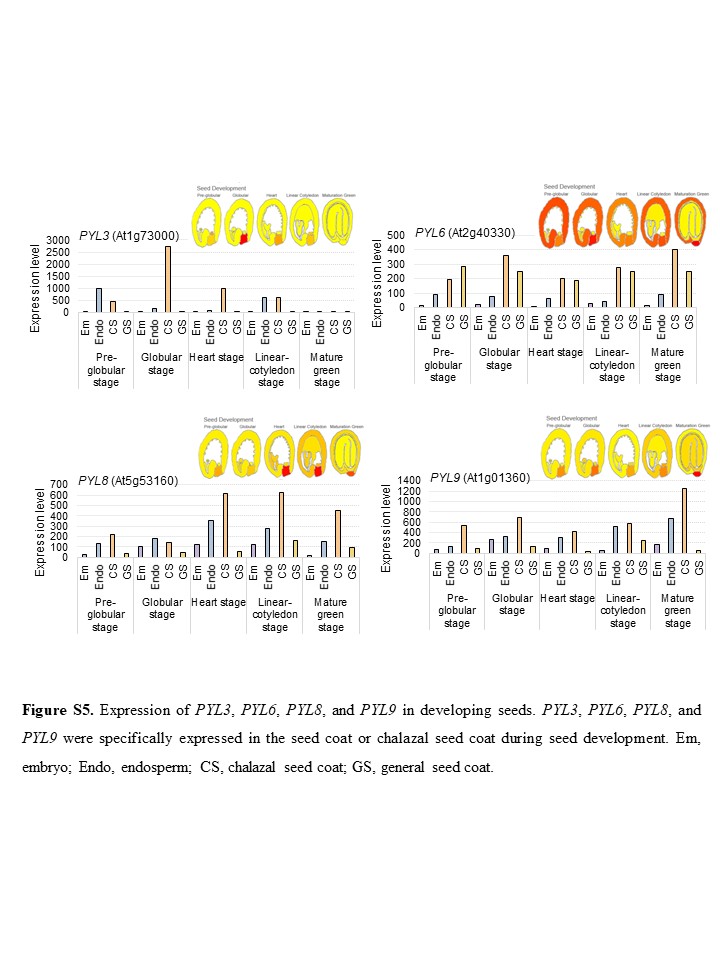

Supplement: Supplementary file 7 [file Image_6.jpeg]
